# Evaluation of the Relationship Between Primary Spontaneous Pneumothorax and Exercise and Return to Previous Activities in Patients Referring to Hospitals of Rasht during 2015–2017

**DOI:** 10.4314/ejhs.v31i3.19

**Published:** 2021-05

**Authors:** Manouchehr Aghajanzadeh, Mohammad Taghi Ashoobi, Seyyed Mostafa Zia Ziabari, Mohammad Reza Asgary, Ramin Ebrahimiyan, Ali Alavi Fomani, Alirza Jafarnejad, Azita Tangestaninejad

**Affiliations:** 1 Department of Thoracic and general Surgery, Guilan University of Medical Sciences, Rasht, Iran; 2 Department of General Surgery, Guilan University of Medical Sciences, Rasht, Iran; 3 Inflammatory Lung Diseases Research Center, Guilan University of Medical Science, iran

**Keywords:** Spontaneous Pneumothorax, Exercise, Return to Sport

## Abstract

**Background:**

The most common cause of primary spontaneous pneumothorax (PSP) is subpleural bleb apical rupture. Little is known about the relationship between PSP and exercise and return to exercise the time. In this study, we tend to investigate the relationship between training and PSP and time of return to exercise and previous activities.

**Method:**

This study was designed as a case series and the sample size included all patients diagnosed with PSP in Razi and Poursina and Aria hospitals of Rasht during 2015–2017 based on inclusion criteria. Variables were analyzed using Fisher's exact test, Chi-square, Mann Whitney U and t-test (p<0.05).

**Results:**

The most common treatment type in patients was transaxillary thoracotomy with pleurodesis with iodine (TTP) in 58.2% and tube thoracostomy and pleurodesis in 41.7%, which was not statistically significant between athletes and non-athletes (p=0.806). Athletes who underwent TTP after four weeks and those treated with tube thoracostomy and pleurodesis after 8–12 weeks were advised to return to their previous activity. Of athletes, 9.5% had a recurrence; of non-athletes, 9.8% had a recurrence. Of athletes, 4.8% did not tolerate returning to their last activity; of non-athletes, 7.3% did not tolerate returning to their previous activity regardless of treatment, and this difference was not significant.

**Conclusion:**

Our study showed no significant difference between clinical manifestations and image findings and the frequency of treatment and complications in both athlete and non-athlete patients. There is no increase in recurrence and intolerance at the time recommended for return to previous activity.

## Introduction

Pneumothorax is defined as the presence of air in the pleural cavity (1). Pneumothorax can occur spontaneously or following direct trauma to the chest (1, 2). Spontaneous pneumothorax is divided into primary and secondary categories (3). Primary spontaneous pneumothorax (PSP) occurs in the absence of any underlying pathological agent(4–6). The most common cause of spontaneous pneumothorax is subpleural bleb apical rupture (7). PSP is more common in men (8). Its incidence in men is 7 to 18 cases per 100,000 population per year and in women 1 to 6 cases per 100,000 people per year (9, 10). Other risk factors include smoking, tall, young men aged 10 to 30 years (11, 12). Moreover, 10% of PSP cases have a positive family history (5, 7). Climate change, including pressure and temperature reductions has also been identified as a contributing factor to PSP (12, 13). Pneumothorax symptoms include sudden onset of severe chest pain in the affected side, dry cough and cyanosis, and even the patient may die of suffocation or acute cardiovascular failure (14). Treatment can vary from inserting a chest tube by connecting to WATER Seal to thoracoscopy and bleb resection and pleurodesis (14). But little has been done about the relationship between PSP and exercise and return to sport time, and there are few reports and surveys in the literature on the relationship and there is disagreement about the main question of when to return to exercise and daily activity (15). Therefore, this study aims to investigate the relationship between exercise and PSP and time of return to exercise and previous activities in Patients Referring to Razi and Poursina and Aria Hospitals of Rasht during 2015–2017.

## Materials and Methods

**Calculation of Sample Size, Data Collection, Implementation and Instruments**: From 2015 to 2017, approximately 314 cases of pneumothorax were referred to Razi, Pooursina and Aria hospitals of Rasht, of which 103 were included in our study. Of 103 patients studied, 21 were athletes and 82 were non-athletes. All of these patients underwent chest intubation after diagnosis of pneumothorax and were visited daily.

**Statistical analysis**: SPSS software version 8 was used for data analysis. Descriptive data were calculated as mean and standard deviation. In this study, variables were analyzed using Fisher's exact test and Chi-square test, Mann-Whitney-U test and t-test (p<0.05).

## Results

In this study, 73.8% of patients were male and 26.2% were female; 20.4% of the patients were athletes and 79.6% were non-athletes; 85.7% were male athletes and 14.2% were female athletes resembling the gender distribution of the disease.

The most common clinical symptom was chest pain (41.7%). The most common exercise was bodybuilding (28.6%), followed by martial arts (19%), track and field and volleyball (14.3%). For 71.4% of athletes, the time interval between onset of symptoms and the last time of exercise was more than seven days and 28.6% had activity during the previous week. Of total subjects, only 3.9% had physical activity at the same time as PSP.

The most common side of PSP was generally the right side (65%). The right side was the most prevalent side in athletes (52.4%) and the right side was the most common side in non-athletes (68.3%), which was not statistically significant (P=0.111).

Moreover, 36.9% of patients were smokers, 19% of athlete patients were smokers and 41.5% of non-athlete patients were smokers. Although the athletes were less smokers, this difference was not significant (P=0.057). Perhaps this difference was significant as the number of samples increased. The most common finding in athlete and non-athlete patients was bleb observed on CT scan, which was not significantly different between athletes and nonathletes (p=0.937) (Table 1).

**Table 1:** percentage frequency of findings in athlete and non-athlete groups

Group	Lung collapse No. (%)	Air leak persistence for more than three days No. (%)	Lung bleb in CT No. (%)	Response to early treatment No. (%)
Athlete	2 (9.5)	3 (14.3)	5 (23.8)	11 (52.4)
Non-athlete	7 (8.5)	12 (14.6)	26 (31.7)	35 (42.7)
Total	9 (8.7)	15 (14.6)	31 (30.1)	46 (44.7)

The most common treatment type in patients was trans-axillary thoracotomy with pleurodesis with iodine (58.2%) and tube thoracostomy and pleurodesis (41.7%). Among athletes, TTP was the most common treatment (47.6%); among non-athletes, TTP was the most common treatment (60.9%), which is not significantly different between athletes and non-athletes based on Fisher exact test (p=0.806) (Table 2).

**Table 2:** percentage frequency of treatments in athletic and non-athletic groups

		Treatment		Total
		
		Trans-axillary thoracotomy with pleurodesis with iodine No. (%)	Tube thoracostomy and pleurodesis No. (%)	
Athlete	N(%)	10 (47.6)	11 (52.4)	21(100)
Non-athlete	N(%)	50 (60.9)	32 (39.1)	82(100)
Sum	N(%)	60 (43.7)	43 (56.3)	103(100)

Among athletes, 47.6% undergoing TTP were recommended to return to their last activity after four weeks and 52.4% undergoing tube thoracostomy and pleurodesis were recommended to their last activity after 8 to 12 weeks. Among non-athletes, 60.9% undergoing TTP were recommended to return to their last activity after four weeks and 39.1% treated conservatively were recommended to return to their previous activity after 12 to 18 weeks.

In this study, 93 patients did not have recurrence, 37.6% underwent tube thoracostomy and the rest underwent TTP. Not all patients with recurrence (10 patients) underwent thoracotomy and all underwent tube thoracostomy. This difference was statistically significant (P<0.0001) in all those who recurred and underwent tube thoracostomy and pleurodesis compared to patients who underwent thoracotomy and did not recurred (Table 3); 96 patients had tolerated treatment, of which 44.8% underwent tube thoracostomy and 55.2% underwent thoracotomy. Of total number of patients who did not tolerate treatment, 28.6% underwent tube thoracostomy and 71.4% underwent thoracotomy. However, thoracotomy was more common among those who did not tolerate the treatment. But this difference was not significant (P=0.464).

**Table 3:** Percentage frequency of recurrence and types of treatments

		Treatment	
		
		Tube thoracostomy and pleurodesis No. (%)	Trans-axillary thoracotomy with pleurodesis with iodine No. (%)
Recurrence	Yes	10 (100)	0
	No	35 (37.6)	58 (62.4)
	**Total**	**45 (43.7)**	**58 (56.3)**
Return to previous	Tolerated	43 (44.8)	53 (55.2)
activity	Not tolerated	2 (28.6)	5 (71.4)
	**Total**	**45 (43.7)**	**58 (56.3)**

Of 21 athletes, 52.4% underwent tube thoracostomy and returned to their previous exercise after 8 to 12 weeks and 18.2% recurred. Of 21 athletes, 10 underwent thoracotomy and returned to exercise after four weeks with no recurrence. Of 82 non-athlete patients, 32 underwent tube thoracostomy, of which 25% recurred after 8 weeks. Comparison of athlete and non-athlete patients who underwent tube thoracostomy and had recurrence using Fisher test showed no significant difference between them (P=0.710) (Table 4). Since there were no recurrences in patients undergoing thoracotomy, no comparison can be made between different subgroups.

**Table 4:** percentage frequency of recurrence in patients of athletic and non-athletic groups after tube thoracostomy

In people recurred after 8 weeks of conservative	Recurrence		Total
		
	Yes N (%)	No N (%)	
Athlete	2(18.2)	9(81.8)	11(100)
Non-athlete	8(25)	24(75)	32(100)
Sum	10(23.3)	33(76.7)	43(100)

Fisher's exact test: P=0.710

Of 21 athlete patients, 47.6% underwent transaxillary thoracotomy and pleurodesis (TTP), of whom 100% tolerated a return to their last activity and no intolerance. Of 82 non-athlete patients, 60.9% underwent TTP, of whom 10% did not tolerate a return to their last activity and 90% tolerated. This difference between athletes and non-athletes was not significant (P=0.578) (Table 5). Of 21 athlete patients, 52.4% underwent tube thoracostomy, who were recommended to return to their last activity after 8 to 12 weeks, one of whom did not tolerate a return to previous activity and had recurrence. Of 82 non-athlete patients, 39.2% underwent tube thoracostomy, of whom 3.1% (one case) did not tolerate return to their last activity after 8 to 12 weeks, and this difference was not significant (P=0.451).

**Table 5:** percentage frequency of tolerating return to previous activity among patients undergoing TTP in people who recurred after 4 and 8 weeks

**Patient**	**Tolerating return to previous activity (4 weeks after operation)**		**Sum**
		
	**Yes (N (%))**	**No (N (%))**	

Athlete	10(100)	0	10(100)
Non-athlete	45(90)	5(10)	50(100)
Sum	55(91.7)	5(8.3)	43(100)

**Patient**	**Tolerating return to previous activity (8 weeks after operation)**		**Total**
	**Yes No (N (%))**	**No (N (%))**	

Athlete	10(90.9)	1(9.1)	11(100)
Non-athlete	31(96.9)	1(3.1)	32(100)
Sum	41(95.3)	2(4.7)	43(100)

Fisher's exact test: P=0.578

Among athletes, recurrence rate was 9.5% and recurrence rate of non-athletes was 9.8%. Overall, this difference was not significant (P=0.998) (Table 6). Of athletes, 4.8% did not tolerate their return to their last activity, and 7.3% of non-athletes did not tolerate their return to their last activity, regardless of the type of treatment, and this difference was not significant (P=0.998).

**Table 6:** percentage frequency of recurrence and tolerance of return to previous physical activities in athlete and non-athlete groups

**Patient**	**Recurrence**		**Total** N (%)

	**Yes** N (%)	**No** N (%)	

Athlete	2 (9.5)	19(90.5)	21(100)
Non-athlete	8(9.8)	74(90.2)	82(100)
Total	10(9.7)	93(90.3)	103(100)

Patient	**Return to previous activity**		**Total** No. (%)
	**Tolerated** No. (%)	**Not tolerated** No. (%)	

Athlete	20 (95.2)	1 (4.8)	21(100)
Non-athlete	76 (92.7)	6 (7.3)	82(100)
Total	96 (93.2)	7 (6.8)	103(100)

## Discussion

Recording PSP cases in athletes is particularly encouraged by practitioners to provide guidelines that improve the diagnosis and treatment of these conditions and they recommend to provide guidelines in determining the safe return time. In this study, we tended to investigate the relationship between exercise and primary spontaneous pneumothorax and the time to return to exercise and previous activities of patients with PSP. The results of our study showed that 73.8% of the participants were male and 26.2% were female. The most common clinical symptom in our patients was chest pain (41.7%). Aghajanzadeh et al studied PSP epidemiology in Rasht during 2009–2014; in their study, 79.9% of patients were male. The most common clinical symptom (53.2%) was chest pain (9). Yoon et al. (2013) examined risk factors for spontaneous pneumothorax; they showed that 65.7% of patients were male, with the most common complaint being chest pain in 35.2% and shortness of breath in 21.7% (2). In addition to clinical findings, our study examined CT scans, with the most common finding in both athlete and non-athlete patients being multiple blebs ranging from 42.7% to 52.4%. Moreover, in our study, 57.3% of patients did not providea description of heavy activity. Yoon et al. noted that 53.4% of patients did not describe of heavy activity. In contrast, Uramoto et al. showed that heavy activity is not a vital, risk factor for predicting spontaneous pneumothorax (8).

The most common part of pulmonary involvement in spontaneous pneumothorax was in the right side of the chest (65%) and only 5.8% of patients had involvement in both sides of the lung. Yoon et al. reported that 3.2% of patients with spontaneous pneumothorax showed both sides of the lung involved (2). Few studies have investigated spontaneous pneumothorax in athletes, most of which were case reports (4–6). In our study, 103 patients with primary spontaneous pneumothorax were evaluated, of whom 21 were athletes (20.4%). In our study, we refer to an athlete who meets the following four criteria: 1) Performing sports to improve performance; 2) Participating actively in sports competitions; 3) Formally recognized in local or regional or national competitions; 4) Exercising and participating in most competitions included daily activity and mental focus. Of the sports studied, bodybuilding (n=6; 5.8%) was the most frequent sports in our study. One of the most important issues in this disease is the high rate of the recurrence despite different treatments available, ranging between 20–50% for primary and 10–20% for secondary cases (9). In contrast, the recurrence rate in our study between athletes and non-athletes ranged from 9.5% to 9.8%, which may be due to the method used to examine patients undergoing CT scans and more patients receiving an intervention. In the present study, the most common treatment type was transaxillary thoracotomy with pleurodesis with iodine, which had the same frequency in both groups. In athletic patients who underwent transaxillary thoracotomy, 100% had tolerated a return to previous activity. Among non-athlete patients, only 10% could not tolerate a return to their previous activity. Of athlete and non-athlete patients who underwent tube thoracostomy and pleurodesis, 18.2–25% had the recurrence. None of our patients who underwent TTP recurred. Overall, 9.5% of the athletes had recurrence and only 4.8% did not tolerate a return to their previous activity. We recommended 47.6% of athletes after four weeks based on TTP treatment received to return to their previous activity and recommended 52.4% to return to their activity after 8 to 12 weeks. As noted earlier, only one case did not tolerate return to previous activity regardless of time interval of return to daily activity. Through a case report and empirical observation, Sean M. Curtin et al. noted that, depending on general conditions and cause of spontaneous pneumothorax, it is possible to decide on sports activities of these athletes and the time to start exercises and sports activity should be limited at least for three weeks and activity must gradually increase at the time of return to exercises under the supervision of the practitioner (6). In a case report by Peter F. Davis et al., a track and field athlete was treated conservatively. The patient showed full recovery six days after onset of symptoms and started light activities after one week and started over the competition after three weeks. This patient had no problem after three months (7).

In conclusion, our study showed that both athlete and non-athlete patients were evaluated based on the same diagnostic and therapeutic indications. There were no significant different in the results derived from clinical presentations and imaging findings and in terms of frequency of treatment and side effects (recurrence and tolerance to return to exercise and the previous activity). Also, the rate of recurrence was not significantly difference between athletes and non-athletes. At the recommended time to return to the previous activity in our study, we did not see any increase in recurrence and intolerance than other studies. Therefore, it can be claimed that return to the previous activity in athletes with spontaneous pneumothorax depends on general conditions and type of treatment, whether conservative or thoracotomy, and regardless of the exercise.
